# Supplementary material for: Migration and division in cell monolayers on substrates with topological defects
Source: Proc Natl Acad Sci U S A. 2023 Jul 18;120(30):e2301197120. doi: 10.1073/pnas.2301197120 (PMC10372565; doi:10.1073/pnas.2301197120)
Supplement: Supplementary file 1 — Appendix 01 (PDF) [file pnas.2301197120.sapp.pdf]

Supporting Information for

Migration and division in cell monolayers on substrates with topological defects

Kurmanbek Kaiyrbekov, Kirsten Endresen, K. Sullivan, Z. Zheng, Yun Chen, Francesca Serra, Brian A. Camley

Brian A. Camley.
E-mail: bcamley1@jhu.edu

This PDF file includes:

Figs. S1 to S15
Legends for Movies S1 to S5

Other supporting materials for this manuscript include the following:

Movies S1 to S5

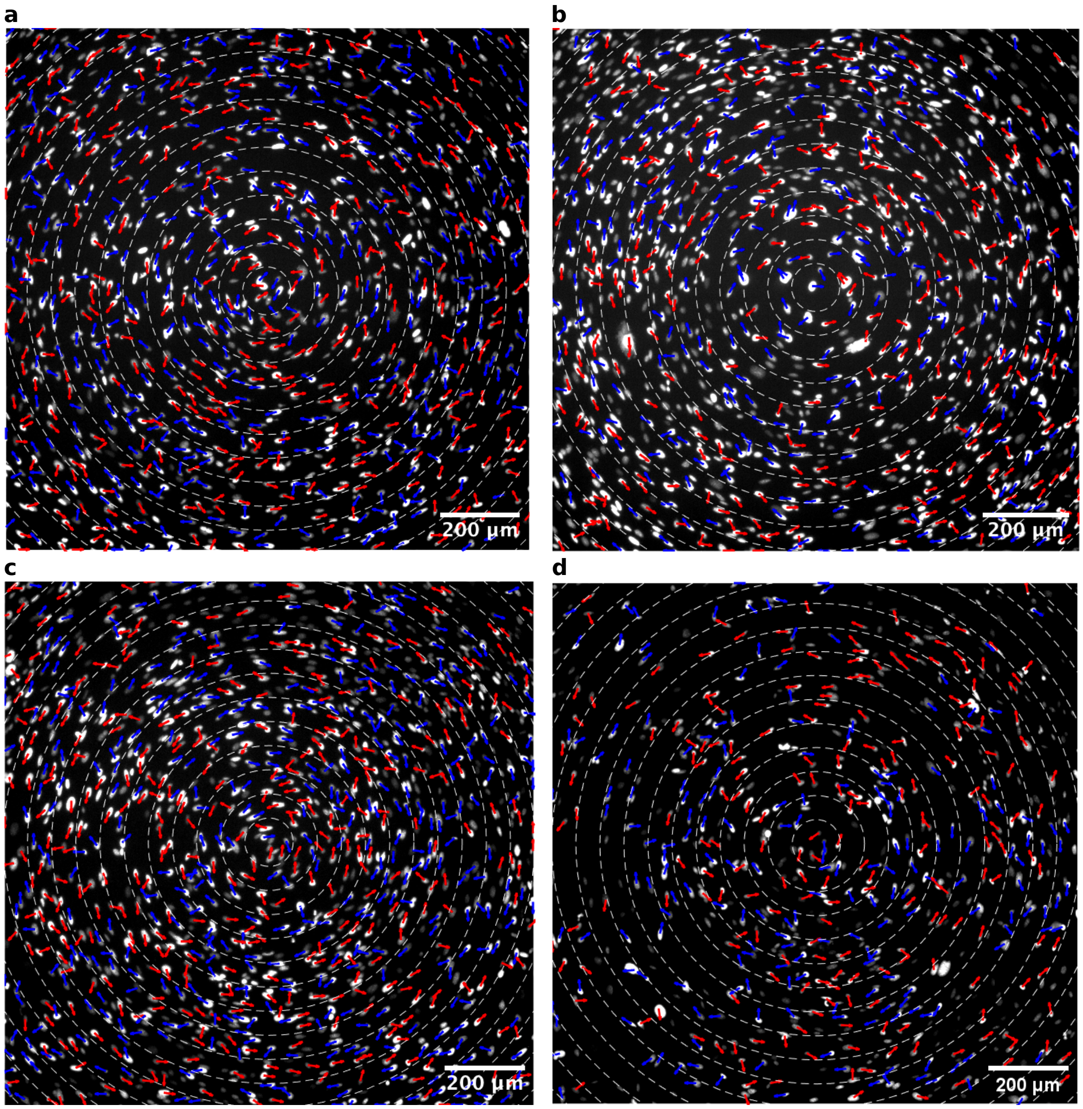

Fig. S1. 3T6 cell nuclei stained with Hoechst 33342 moving in the vicinity of +1 defects. White dashed lines indicate the location of h=1.5 μm ridges. Scale bar is 200 μm. Arrows represent the direction of cells' motion within one hour, identified using TrackMate, with red arrows indicating counterclockwise motion and blue arrows indicating clockwise motion. Densities are (**a**) 560 cells/mm^2, (**b**) 630 cells/mm^2, (**c**) 690 cells/mm^2, and (**d**) 410 cells/mm^2

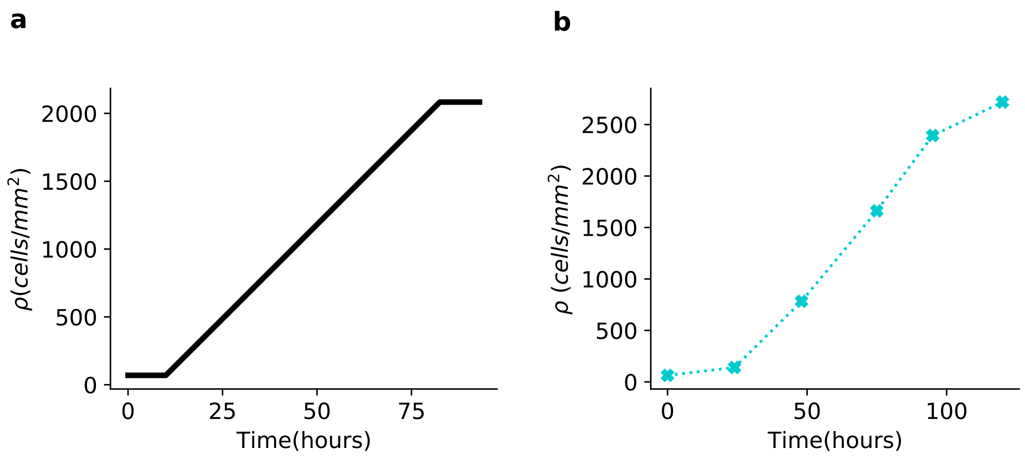

Fig. S2. Growth curves show cell number increases over time and then begins to saturate. **a** Simulation. Note this growth curve is imposed by our approach, and chosen to roughly match the experiment (see Methods). **b** Experiment on 3T6 fibroblasts, conducted as detailed in Methods.

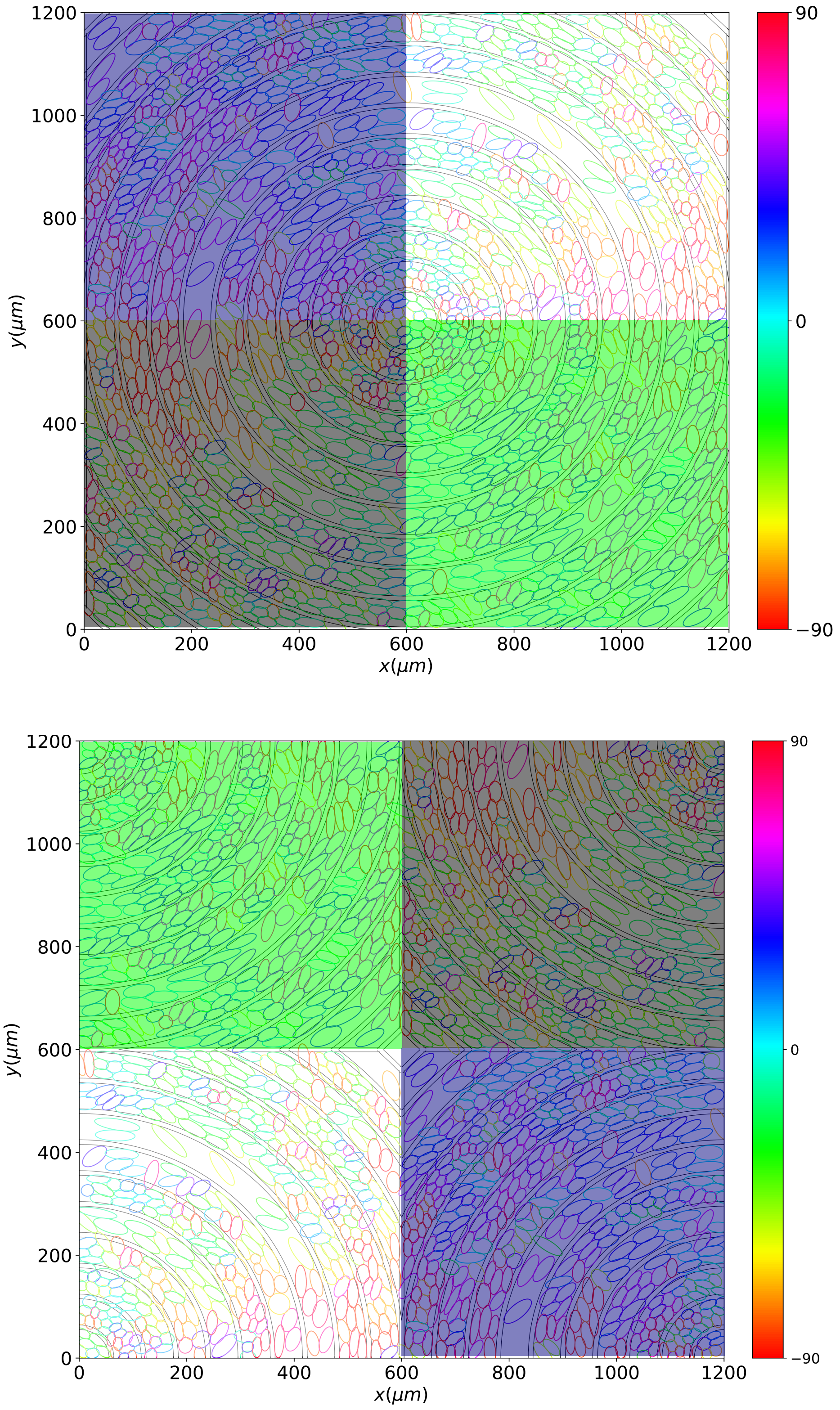

Fig. S3. Simulation box has dimensions of 1200x1200 μm^2 and periodic boundary conditions. If +1 defect is centered then -1 defect is at the corners of simulation box. The figure demonstrates of rearrangement of quadrants of the simulation box with centered +1 defect (top) that centralizes -1 defect (bottom). Color bars indicate the angle between major axis of cells and x-axis.

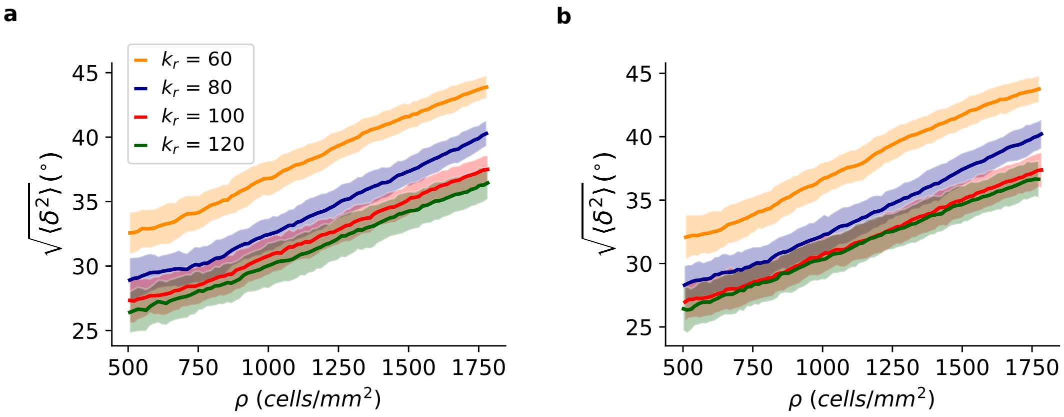

Fig. S4. Simulation: RMSDs $\sqrt{\langle\delta^2\rangle}$ for different ridge strengths when deviations are computed with respect to the tangent line of a ridge point closest to a cell for +1 (**a**) and -1 (**b**) defects. To determine the closest ridge point, we divided contiguous ridges into equally spaced discrete points. The distance between successive points was set to $1\ \mu m$. Then, for each cell, we found closest point to its center of mass and computed deviation δ of the cell orientation from tangent line of the closest ridge point.

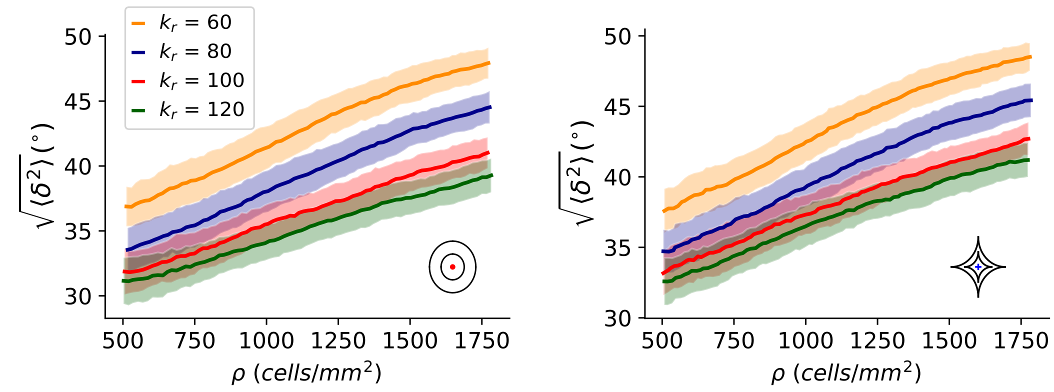

Fig. S5. Simulation: RMSDs $\sqrt{\langle\delta^2\rangle}$ for different ridge strengths when cells are randomly selected to divide, i.e. $p_i = 1/N$, and in the absence of motility energy (i.e. $k_{\mathrm{move}} = 0$) for +1 (left) and -1 (right) defects.

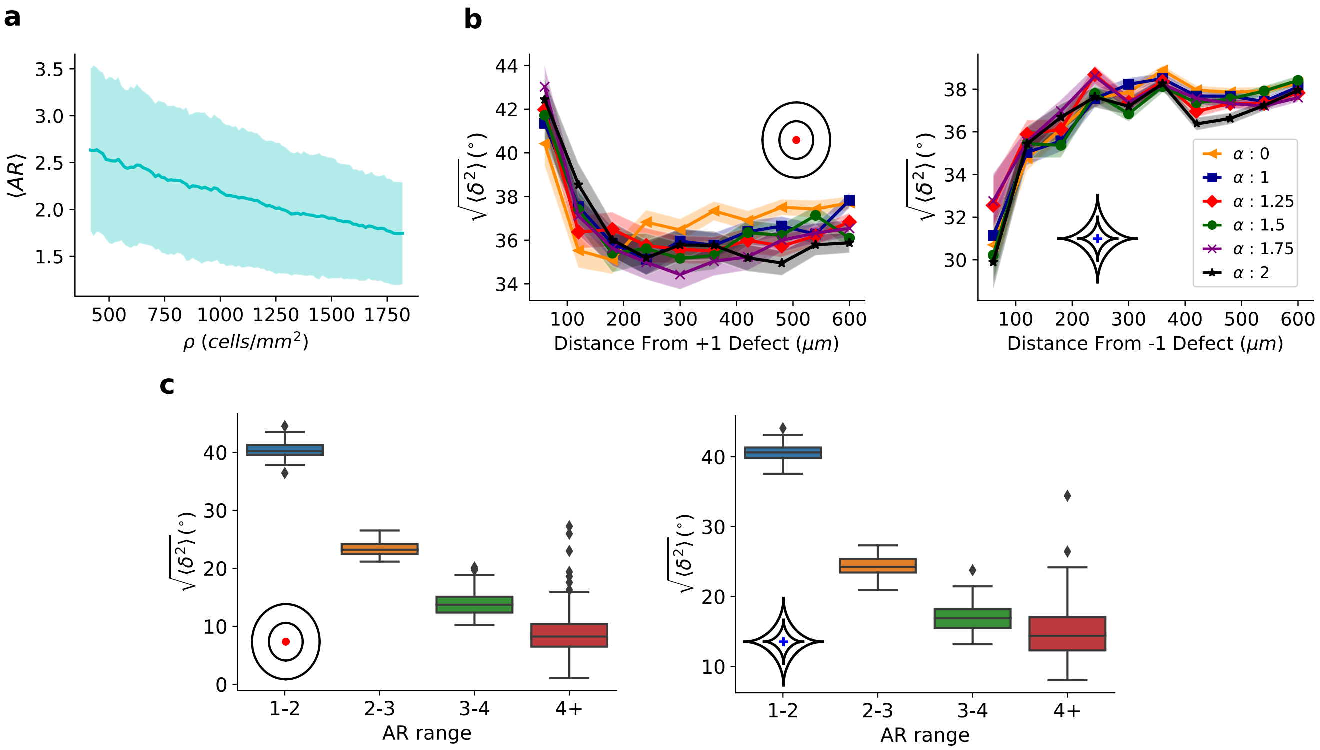

Fig. S6. Simulation: Evolution of cell aspect ratios and dependence of RMSDs $\sqrt{\langle\delta^2\rangle}$ on aspect ratios and distance to defect center. Ridge strength is set to default value of $k_r = 120$. **a**, Average aspect ratio as a function of cell density for single simulation. Averaging is done over cells and shaded area is a standard deviation. **b**, RMSDs near positive (left) and negative (right) defects for different sensitivities of division probability to shape α. **c**, Boxplots of average RMSDs for cells with different aspect ratios for positive (left) and negative (right) defects. Averaging is done over cells within 600 μm from the respective defect core. There are 100 data points for each aspect ratio range representing 100 simulations. The data are taken at a density of $\sim 1600\ \mathrm{cells/mm^2}$ and outliers are shown in black diamonds.

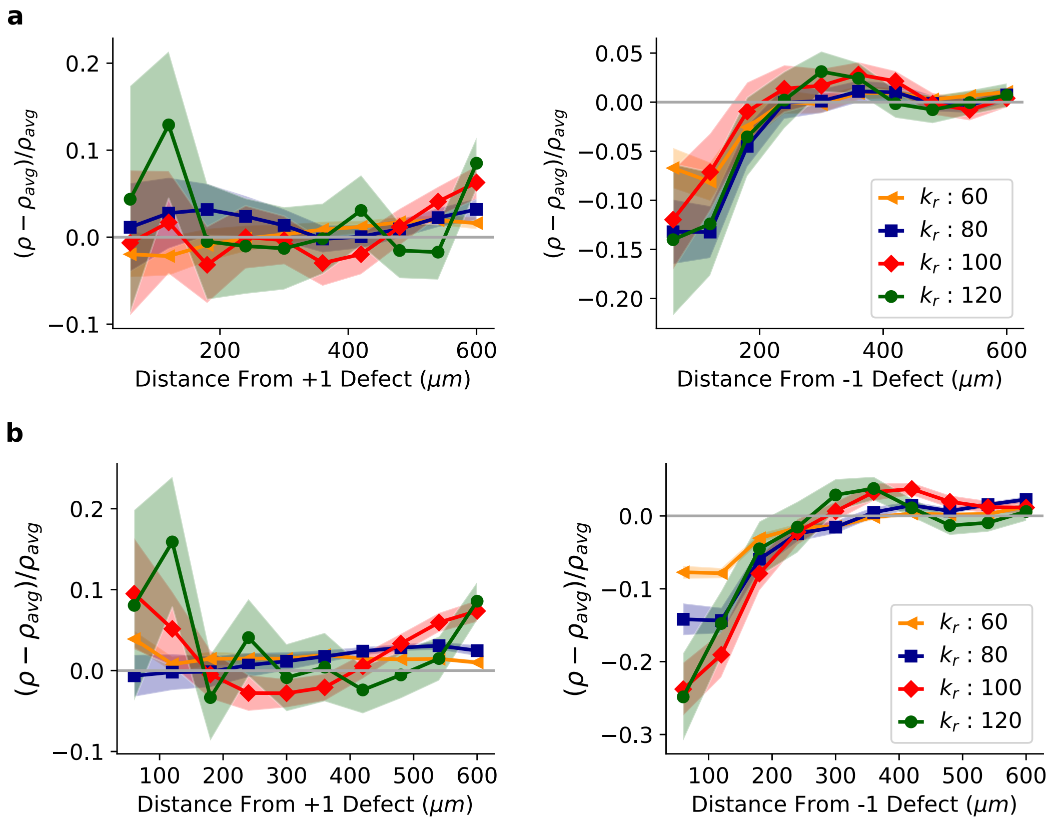

Fig. S7. Simulation: Density profiles for different ridge strengths when cells are randomly selected to divide, i.e. $p_i = 1/N$. **a**, Densities near +1 (left) and -1 (right) defects without motility energy ($k_{\rm move} = 0$). **b**, Densities near +1 (left) and -1 (right) defects with motility energy ($k_{\rm move} = 1T/\mu m$).

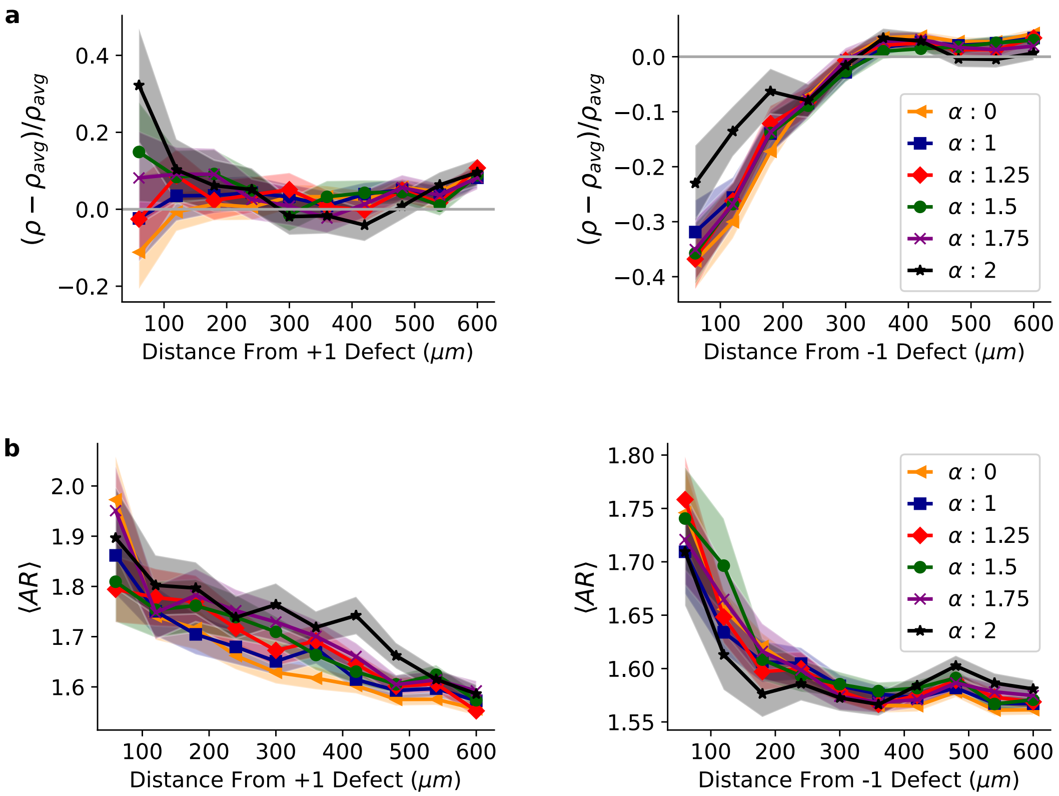

Fig. S8. Simulation: Density and aspect ratio profiles in the absence of motility energy ($k_{\mathrm{move}} = 0$) and ridge strength of $k_r = 120$ for different sensitivity of division probability to shape α. **a**, Densities near positive (left) and negative (right) defects. **b**, Aspect ratios near positive (left) and negative (right) defects.

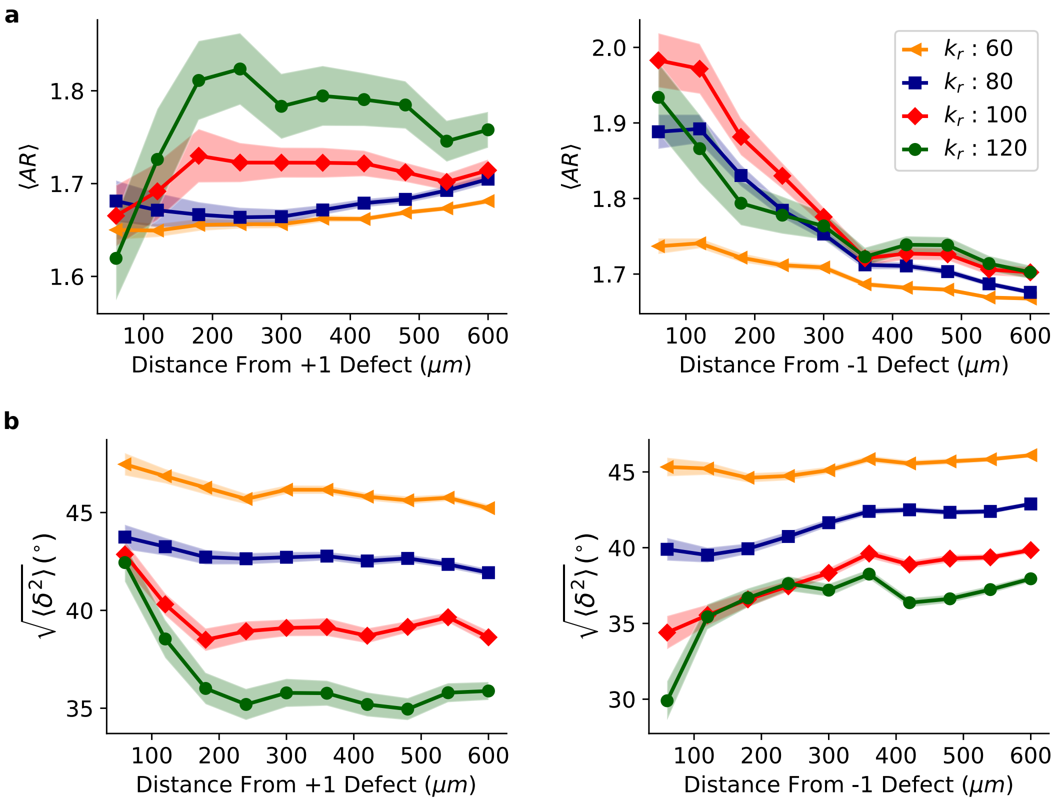

Fig. S9. Simulation: Aspect ratio (**a**) and RMSD (**b**) profiles for different ridge strengths near +1 (left) and -1 (right) defects at the end of simulation. Here $\alpha = 2$ and $k_{\rm move} = 1T/\mu m$

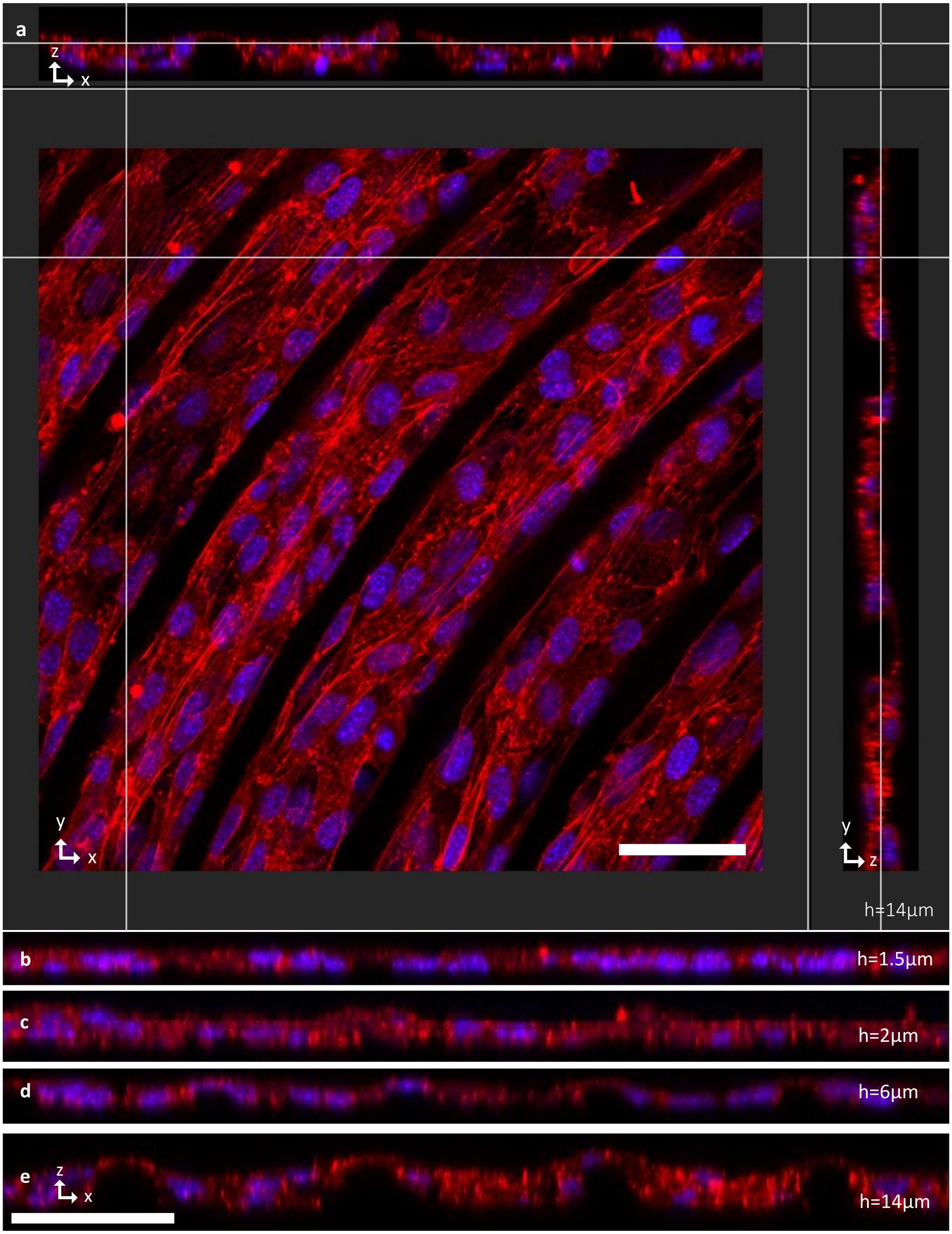

Fig. S10. Experiment: Confocal imaging of 3T6 cells to show 3D structure. **a**, Confocal image of nuclei (stained with Hoechst 33342) and actin filaments (stained with Phalloidin-iFluor 594 conjugate). This shows three orthogonal views, the top-down view and two side views, with the location of the cross-sections shown by the crosshairs. The scale bar is 50 μm. **b**, Cross-section from a h=1.5μm sample, showing the nuclei lying in a flat monolayer. Scale bar for **b**-**e** is 50 μm. **c**,**d** Cross-section from h=2μm and h=6μmsamples, respectively. As the ridge height increases, the monolayer becomes more disrupted, but the nuclei still occupy the same plane. **e**, Cross-section from a h=14μm sample. In this case the monolayer becomes more undulated, and the nuclei can be seen at different heights, indicating that the cell environment is becoming more 3-dimensional.

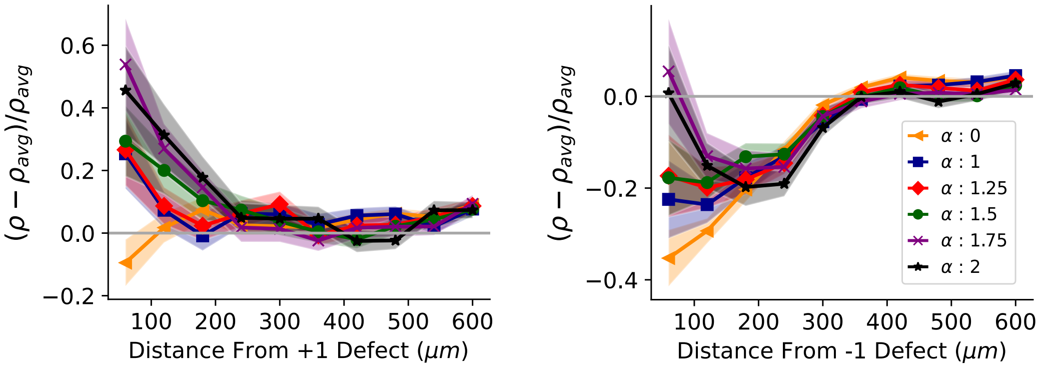

Fig. S11. Simulation: Density profiles for different sensitivities of division probability to shape α near positive (left) and negative (right) defects when ring spacing is changed to make the areas enclosed within the innermost region of the +1 and -1 defects closer to one another. To match areas in a better way, we decreased spacing between ridges of incomplete rings from 55 μm to 50 μm such that resulting inner area at the core of the -1 defect increases from $\sim 3300\ \mu m^2$ to $\sim 9500\ \mu m^2$. Note that the area of the inner ring of the +1 defect is 11310 μm^2.

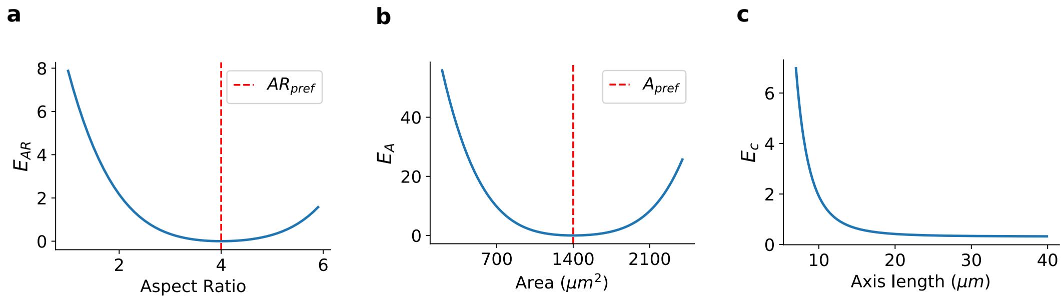

Fig. S12. Geometric energies that penalize deviations from preferred shape and size. **a**, Quartic aspect ratio penalty for preferred aspect ratio $AR_{\text{pref}} = 4$. **b** Quartic area deviation penalty energy for preferred area $A_{\text{pref}} = 1400\ \mu m^2$. **c**, Core energy as a function of one of the axis lengths when the other axis is set to $15\ \mu m$.

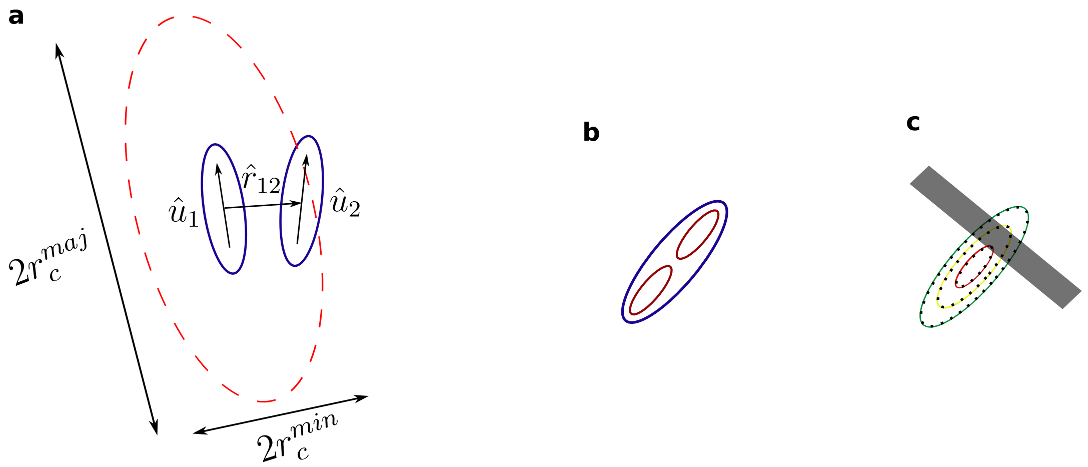

Fig. S13. Illustrations for cell-cell interaction, division and cell-ridge interactions. **a**, Schematic of the Gay-Berne interaction cutoff region for cell 1 (with orientation $\hat{u}_1$) shown with red dashed ellipse. The ellipse has major axis length $2r_c^{\mathrm{maj}}$ and minor axis length $2r_c^{\mathrm{min}}$. Cell 2 with orientation $\hat{u}_2$ is within interaction range. **b**, Illustration of a parent cell (blue) dividing into two daughter cells (red). **c**, Sketches of three ellipses with dimensions (a, b), (2a/3, 2b/3) and (a/3, b/3) that are used to sense ridge shown by gray area. The dark dots within shaded ridge area contribute to cell-ridge overlap penalty.

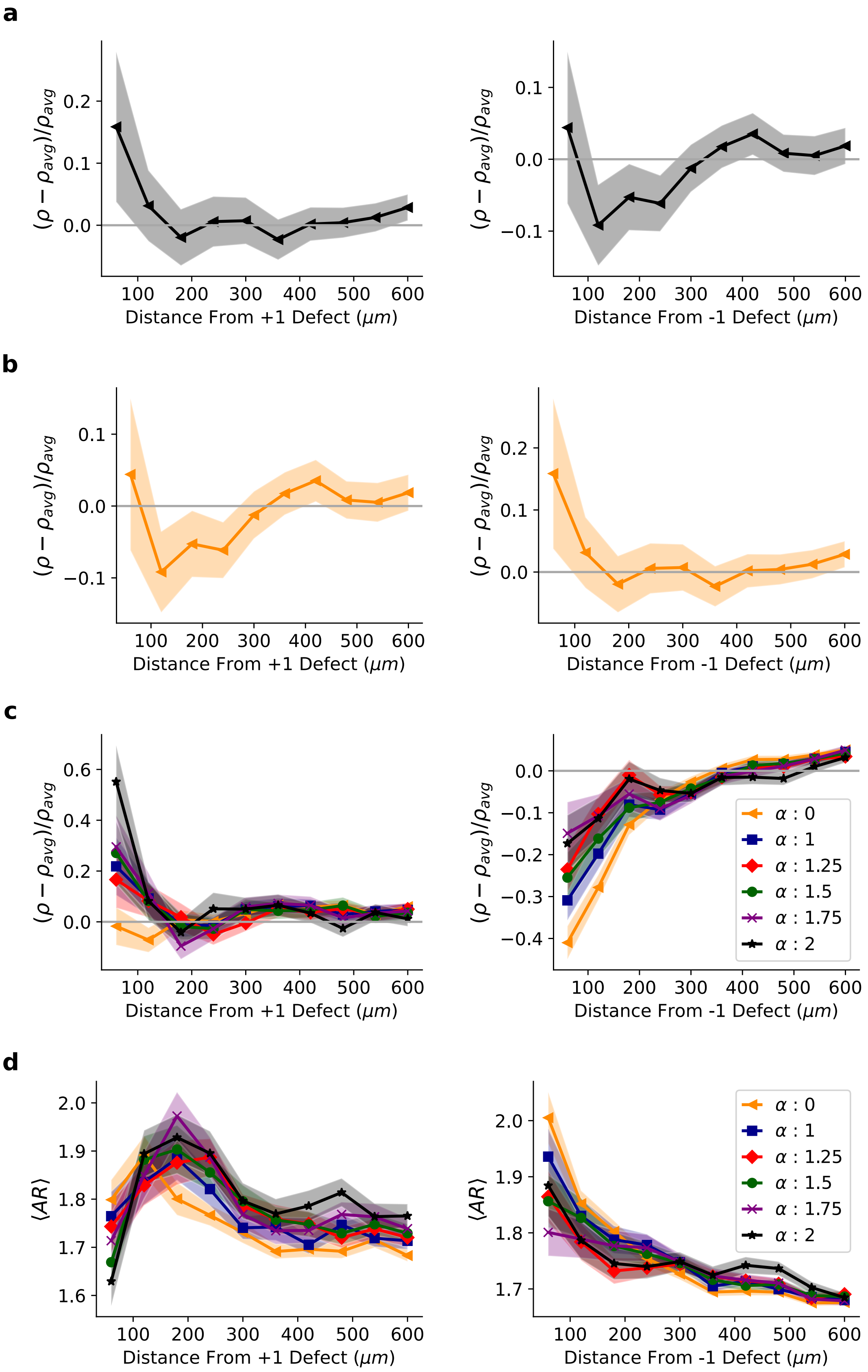

Fig. S14. Simulation: Density and aspect ratio profiles when initial conditions are swapped. **a**, Default initial density profiles at the start of simulation for +1 defect (left) and -1 defect (right). **b**, Swapped initial conditions. End state densities (**c**) and aspect ratios (**d**) near +1 defect (left) and -1 defect (right) when initial conditions are swapped for different values of shape sensitivity α. Here $k_r = 120$.

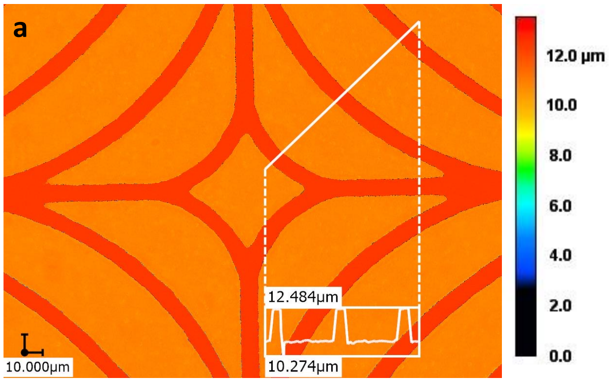

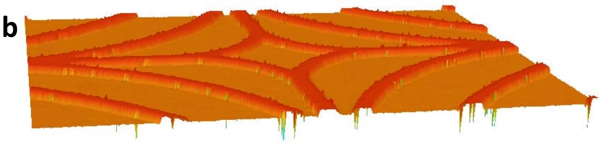

c

Nominal Height	Measured Height
$1.5\mu m$	$1.5 \pm 0.1\mu m$
$2\mu m$	$2.0 \pm 0.1\mu m$
$6\mu m$	$5.8 \pm 0.5\mu m$
$14\mu m$	$14 \pm 2\mu m$

Fig. S15. Experiment: topography of the pattern. **a** Top-down view of h=1.5μm PDMS acquired with the laser scanning microscope. The color represents height. **b** 3D rendering of the same image, with the colors also representing height. Here, the spikes below the surface at the edges of the ridges are artifacts resulting from scattered light. (c) Table of the measured heights (mean and standard deviation) of the PDMS samples. The ridge heights of the h=1.5μm, 2μm, and 6μm samples were measured using the laser scanning microscope, and the h=14μm sample was measured by viewing the cross section of the PDMS with the optical microscope with a 50X objective.

Movie S1. Cell movement around +1 defect. 8 hour experimental video from fluorescence microscopy showing nuclei stained with Hoechst 33342 which have been tracked using TrackMate in ImageJ. h=1.5μm and ridge spacing is 60μm.

Movie S2. Time evolution of simulation with centered +1 defect from 100 cells to 3000 cells in 93 hours. Cells are colored according to angle they make with x-axis as in Fig. 2

Movie S3. Same trajectory as Movie 2, but recentered such that -1 defect is at the core.

Movie S4. Simulation showing mixing of cells when ridge strength k_r is set to 60. At the start of simulation cells are colored according to their radial location. If a cell is initially within the first (innermost) ring, it is colored orange, cells between the first and second ring are colored black, etc. All descendants of cell inherit color from their parent (or equivalently root) cell. We can see that cells that origins at different locations eventually cross ridges and mix.

Movie S5. Same as Movie 4, but for the ridge strength $k_r = 120$. In this case the overwhelming majority of cells do not cross ridges.
